# Autonomic neuropathic symptoms in patients with diabetes: practical tools for screening in daily routine

**DOI:** 10.1186/s13098-023-01036-7

**Published:** 2023-04-26

**Authors:** Ana Raquel Souza de Azevedo Vieira, Lara Benigno Porto-Dantas, Flaviene Alves do Prado Romani, Patrícia Souza Carvalho, Rodica Pop-Busui, Hermelinda Cordeiro Pedrosa

**Affiliations:** 1 Unit of Endocrinology of the Regional Hospital of Taguatinga and Research Center of the Foundation for Education and Research in Health Sciences, Secretariat of Health of the Federal District, Brasilia, Brazil; 2 Division of Metabolism, Endocrinology & Diabetes, Department of Internal Medicine, University of Michigan, Ann Arbor, MI USA

**Keywords:** Diabetic autonomic symptoms, Autonomic neuropathy, Sudomotor dysfunction, Screening tests

## Abstract

**Background:**

Diabetic autonomic neuropathy (DAN) is a frequent complication in people with diabetes whose screening is often neglected. This study aimed to evaluate DAN through practical tools in people with diabetes in a referral center for diabetes treatment.

**Methods:**

DAN symptoms and severity were assessed using the Survey of Autonomic Symptoms (SAS) via digital application (app) in patients attended from June 1, 2021, to November 12, 2021. SAS scoring for DAN was performed using established validated cutoffs. The adhesive with cobalt salt color indicator (Neuropad™) was used as a measure of sudomotor dysfunction. Demographical and clinical data were also collected.

**Results:**

Data from 109 participants, 66.9% T2DM, 73.4% female, with a median age of 54.00 (± 20.00) years, were analyzed. Symptomatic DAN was present in 69.7% of participants and was associated with older age (p = 0.002), higher HbA1c (p = 0.043), higher abdominal circumference (p = 0.019), higher BMI (p = 0.013), more likely to have metabolic syndrome (MS) with a 10-fold increased risk, and more frequent association with diabetic peripheral neuropathy (p = 0.005). Sudomotor dysfunction was found in 65 participants with positive Neuropad™ detected in 63.1% of them.

**Conclusion:**

The use of SAS through an app proved to be a practical and easy-to-use instrument to document symptoms of DAN in busy clinical practice. The high frequency of symptoms draws attention to the importance of screening this underdiagnosed diabetes complication. The risk factors and comorbidities associated with symptomatic DAN highlight the patients’ phenotypes linked to MS that should be targeted for DAN evaluations in larger samples in the community.

**Supplementary Information:**

The online version contains supplementary material available at 10.1186/s13098-023-01036-7.

## Introduction

Diabetic autonomic neuropathy (DAN), an underdiagnosed diabetes complication, is defined as “an autonomic nervous system disorder that results from changes caused by diabetes or prediabetes, after exclusion of other possible causes” [1, 2]. It is due to damage to the small unmyelinated type C nervous fibers [3], and the clinical picture may vary from subclinical to symptomatic states, requiring specific tests for diagnosis confirmation [1, 2, 4]. Several risk factors have been shown to contribute to DAN development, including diabetes duration, poor glucose control, obesity, dyslipidemia, high blood pressure, and microvascular complications [3, 4].

Although cardiovascular autonomic neuropathy (CAN) is the most studied and clinically relevant DAN [1, 4, 5], other forms of DAN may also be present, such as gastrointestinal (gastroparesis and enteropathy - diarrhea, colonic hypomotility, constipation), urogenital (cystopathy, neurogenic bladder, male erectile dysfunction, female sexual dysfunction), sudomotor (gustatory sweating, distal hypohidrosis/anhidrosis), unnoticed hypoglycemia and pupillary dysfunction [1, 2, 6].

Gold-standard diagnostic tests for DAN, while sensitive and specific, require sophisticated expensive equipment and, in general, quite cumbersome for patients and providers. Thus, simpler instruments, such as patient-reported outcome questionnaires, have been developed to identify DAN as more practical and lower-cost modalities [5]. One such instrument is the “*Survey of Autonomic Symptoms*” (SAS) validated by Zilliox et al. [6] as a simple, specific, and sensitive for detecting symptomatic DAN in several domains: orthostatic adaptation, sudomotor, vasomotor, gastrointestinal, urinary and sexual, the latter being restricted to males. This screening instrument is practical and has good sensitivity and high specificity when compared to other more complex methods validated for the study of DAN symptoms (Autonomic Symptom Profile - ASP; Composite Autonomic Symptom Scale – COMPASS; Composite Autonomic Scoring Scale – CASS; Quantitative Sudomotor axonal Reflex - QSART) [6].

Among the manifestations of DAN, sudomotor dysfunction has been shown to be a risk factor for cardiovascular disautonomy [5], diabetic peripheral neuropathy (DPN) [7], foot complications and preulcerative lesions [8] in people with diabetes. Several screening devices have been developed to assess this complication; they are simple, practical, and low-cost, such as the already validated adhesive with cobalt salt color indicator Neuropad™ [7], which is easily applied to the plantar skin of the forefoot region bilaterally to verify post ganglionic small fiber C sudomotor dysfunction [9].

Considering that DAN is an underdiagnosed diabetes complication despite its great impact on the quality and survival of patients with diabetes mellitus (DM), we aimed to assess symptoms of DAN combined with a digital app and an inexpensive and easy-to-use skin test for sudomotor dysfunction to evaluate DAN in people with diabetes followed at a large public referral center in Brazil.

## Methods

### Design and setting

This was a cross-sectional study conducted at the Endocrinology Unit of the Taguatinga Regional Hospital/Research Center (UENDO-POLO-HRT), which is a public referral hospital for diabetes treatment in the Midwest region of Brazil, and at a primary care center of the same health district that supports hospital care. The study was run from June 1, 2021, to November 12, 2021, during the COVID-19 pandemic. The sample involved patients with type 1 (T1DM) or type 2 diabetes (T2DM), at least six years of duration and over 18 years of age, who attended routine appointments.

Participants were enrolled during regular clinical attendance and on a consecutive basis. The exclusion criteria were cognitive limitation to understand and answer the SAS; neurological sequelae of stroke or neurodegenerative diseases (Parkinson’s disease, dementia, Alzheimer’s disease) due to the possibility of association with peripheral and central neuropathies; use of topiramate or beta-blockers, GLP1 analogs (due to adverse effects similar to gastrointestinal DAN symptoms); pregnant women; and patients with glycated hemoglobin (HbA1c) ≥ 11%.

### Demographic and clinical variables

General data such as age, sex (male or female), type of DM, duration of diagnosis and type of treatment were collected through medical records or in the form of direct questioning. Weight and height were measured using a scale and stadiometer, in addition to the mean of the duplicated measurement of the abdominal circumference (AC). Body mass index (BMI) was calculated (kg/m^2^).

Laboratory data were collected in the last 12 months, including the mean of two HbA1c results by certified methods (HPLC or turbidimetry), lipid profile, and serum creatinine (both performed in a certified laboratory).

The presence of metabolic syndrome (MS) was verified through the criteria of the International Diabetes Federation (IDF), which defines this condition by the presence of altered abdominal circumference (AC) men > 90 cm and women > 80 cm, and two or more other criteria: triglycerides ≥ 150 mg/dL, HDL ≤ 40 mg/dL for men and ≤ 50 mg/dL for women; blood pressure ≥ 135 × 80 mmHg and fasting glucose ≥ 100 mg/dL [10].

Microvascular complications were assessed using medical record data. Ophthalmic, kidney disease and diabetic peripheral neuropathy (DPN) screenings are requested annually and routinely for patients with type 2 DM (T2DM) and after five years of diagnosis for type 1 DM (T1DM) [1, 2, 11–13].

Diabetic retinopathy (DR) was evaluated with fundoscopy or retinal mapping data performed by an ophthalmologist in the last 12 months and graded as nonproliferative, proliferative diabetic retinopathy or macular edema [11, 12].

Diabetes kidney disease (DKD) was defined based on the albumin/creatinine ratio (ACR) obtained from either random or timed 12-hour urine samples and on the estimated glomerular filtration rate (eGFR). Albuminuria was defined as albumin creatinine ratio (ACR) ≥ 30 mg/g [11, 12] in at least two samples from three collections at an interval of three to six months. The eGFR was calculated using the CKD-EPI equation [14], as recommended by KDIGO [15] and other societies [11, 12].

DPN was identified from medical records data using previously validated criteria [16, 17], which also includes screening for peripheral arterial disease (PAD) and risk of foot ulceration [18]. Patients who did not have an updated DPN exam were scheduled for evaluation for both small fibers (pain and temperature, using a toothpick and a 128 Hz cold tuning fork handle, respectively) and large fibers (vibration perception, using a 128 Hz tuning fork and the Achilles reflex, with a Babinski hammer) [11–13] according to the modified Neuropathy Disability Score (NDS) [19] already validated in Brazil [16, 17]. The 10 g Semmes‒Weinstein monofilament (SORRI®-Bauru-São Paulo) was used to screen for the neuropathic risk of ulcer and amputation, as recommended by scientific societies [11–13] and the International Working Group on the Diabetic Foot (IWGDF) [19]. For DPN, the definition followed the validated assessment by Abbot et al. [20].

### Evaluation of DAN symptoms

The presence of DAN symptoms was assessed with the “*Survey of Autonomic Symptoms*” (SAS) validated by Zilliox [6] (Table 1). The SAS consists of an 11-item symptoms questionnaire for women and 12 for men across several domains of autonomic function: adaptation to orthostatism, sudomotor, vasomotor, gastrointestinal, urinary, and sexual function; the latter only applied for males (Table 1). Each item on SAS requires a *yes* or *no* response. We considered a cutoff point ≥ 3 positive answers to define the presence of DAN symptoms [6]. The severity of symptoms corresponding to each question on SAS was evaluated on a Likert scale from 1 (the least severe) to 5 (the most severe) [6]. The sum of points on the Likert scale provides the total symptom score (TIS).

**Table 1:** Survey of Autonomic Symptoms (SAS)

*Survey of Autonomic Symptoms* (SAS)	Q1a. Have you had any of the following health symptoms during the past 6 months? Yes = 1 No = 0		Q2b. If you answered *yes* in Q1, how much would you say the symptom bothers you? 1 = Not at all 2 = A little 3 = Some 4 = A moderate amount; 5 = A lot				
1. Do you have lightheadedness?	1	0	1	2	3	4	5
2. Do you have a dry mouth or dry eyes?	1	0	1	2	3	4	5
3. Are your feet pale or blue?	1	0	1	2	3	4	5
4. Are your feet colder than the rest of your body?	1	0	1	2	3	4	5
5. Is sweating in your feet decreased compared to the rest of your body?	1	0	1	2	3	4	5
6. Is sweating in your feet decreased or absent (for example, after exercise or during hot weather)?	1	0	1	2	3	4	5
7. Is sweating in your hands increased compared to the rest of your body?	1	0	1	2	3	4	5
8. Do you have nausea, vomiting, or bloating after eating a small meal?	1	0	1	2	3	4	5
9. Do you have persistent diarrhea (more than 3 loose bowel movements per day)?	1	0	1	2	3	4	5
10. Do you have persistent constipation (less than 1 bowel movement every other day)?	1	0	1	2	3	4	5
11. Do you have leaking of urine?	1	0	1	2	3	4	5
12. Do you have difficulty obtaining an erection (men)?	1	0	1	2	3	4	5

### SAS translation and cultural adaptation to the brazilian portuguese (SAS-QSA)

In Portugal, Valente et al. [21] applied SAS to verify the prevalence of dysautonomic symptoms in patients with T2DM, and it was named “*Questionário de Sintomas Autonômicos*” (QSA). For the present study, SAS underwent translation and cultural adaptation to the Brazilian Portuguese at our unit according to an appropriate methodology. The average Content Validity Index (CVI) of 0.942 was reached, which indicated high instrument reliability, while the Cronbach’s alpha coefficient was 0.52, indicating a moderate value (0.41–0.60) (data not published) [22, 23]. In Brazilian Portuguese, SAS was also named QSA [**Supplementary material**].

### App development for using brazilian SAS

A digital application (app) was specifically designed with the Brazilian Portuguese SAS version (QSA), which was used for the data collection instrument. This was developed in Progressive Web App (PWA) format and with Google’s Angular Framework connected to an Applications Protocol Interface (API) developed in Hypertext Preprocessor (PHP) with a relational database management system based on a query language (MySQL). The time to perform the application of SAS through the app achieved a mean time of 10 min.

### Assessment of sudomotor dysfunction neuropathy applying neuropad™

Neuropad™ can be used to evaluate sudomotor function which is under control of the post ganglionic cholinergic sympathetic small type C fiber innervation [24]. It consists of an adhesive pad containing cobalt salts that is attached to the plantar aspect of the foot and changes color from blue to pink within 10 min [9]. The result is considered abnormal if there is no color change or if the blue color blends with the pink color, which suggests the presence of peripheral autonomic neuropathy [9, 24, 25]. The test has good reproducibility [26]. It was applied to the group of patients who responded positively to questions 5 and/or 6 and/or 7, which evaluate sudomotor symptoms.

### Evaluation of postural hypotension

For patients with a positive answer to question 1, which is suggestive of cardiovascular autonomic dysfunction, postural hypotension was evaluated. After five minutes in the supine position, blood pressure was measured twice with a sphygmomanometer and the means of systolic blood pressure (SBP) and diastolic blood pressure (DBP) were calculated. Subsequently, after 3 min of orthostasis, two other measurements were performed, and the means of systolic and diastolic pressures were again calculated. Patients who had a drop in SBP ≥ 20 mmHg or DBP ≥ 10 mmHg were diagnosed with postural hypotension [1, 26–28].

### Statistical analysis

Descriptive statistics were used to present the clinical and demographic variables of the participants. Absolute and relative frequencies, means and standard deviations (SDs), medians and interquartile ranges were used as appropriate. Differences in the distribution of categorical variables were analyzed using the chi-square test. The Kolmogorov–Smirnov test was used to verify whether continuous variables were normally distributed. Parametric continuous variables were evaluated with a t test, and nonparametric variables were evaluated with the Mann–Whitney test.

Logistic regression analysis was used to calculate the risk ratio of factors associated with the presence of dysautonomic symptoms according to SAS ≥ 3 points. For this analysis, the main predictor variables were inserted (according to the clinical findings and the result of the bivariate analysis), which were selected to obtain the model with the best fit. The selection of variables was performed using the backward stepwise method with a likelihood ratio. Statistical significance was defined as a p value < 0.05. Analyses were performed using the Statistical Package for the Social Sciences (SPSS), version 22.0.

### Ethical Standards

This work was approved by the local Research Ethics Committee under the Certificate of Presentation for Ethical Appreciation (CAAE) number 45280821.5.0000.5553/Approval Number 4.746.304. Written informed consent was obtained from all patients.

## Results

A total of 109 patients were included in the study, and the median age was 54.00 (± 20.00) years. The sex distribution was 73.4% (n = 80) women and 26.6% (n = 29) men. According to the type of diabetes, 33.1% (n = 36) presented T1DM, and 66.9% (n = 73) presented T2DM. The clinical data of the participants are shown in (Table 2).

**Table 2:** Clinical data of participants comparing the presence of DAN symptoms (SAS ≥ 3) vs. absence of DAN symptoms (SAS < 3)

Characteristics	Overall (n = 109)	SAS ≥ 3 (n = 76)		SAS < 3 (n = 33)	Value of p* ^#^
Age ^a^	54.00 (± 20.00)		56.00 (± 19.80)	48.00 (± 22.5)	0.002**
HbA1c ^a^	7.70 (± 2.25)	7.97 (± 2.28)		7.50 (± 1.10)	0.043**
Time of DM ^a^	14.00 (± 16.00)	14.00 (± 16.00)		16.00 (± 15.50)	1.000
Use of insulin ^b^	52 (± 47.7)	31 (± 40.9)		21 (± 63.6)	0.028**
AC ^c^	101.01 (± 14.37)	103.13 (± 14.58)		96.15 (± 12.80)	0.019**
BMI ^a^	28.73 (± 6.70)	29.54 (± 7.5)		27.56 (± 5.10)	0.013**
Dyslipidemia ^b^	72 (66.1)	55 (72.4)		17 (51.5)	0.035**
HDL ^c^	48.45 (± 15.11)	47.43 (± 14.81)		50.92 (± 15.84)	0.333
Triglycerides ^a^	137.00 (± 100.50)	151.00 (± 101.50)		102.00 (± 7.00)	0.072
SBP ^c^	133.76 (± 19.23)	135.73 (± 19.44)		129.21(± 18.20)	0.104
DBP ^c^	78.52 (± 11.32)	78.44 (± 11.86)		78.69 (± 10.15)	0.916
MS ^b^	95 (87.2)	72 (94.7)		23 (69.7)	0.001**
Overweight	46 (42.2)	30(39.5)		16 (48.5)	0.381
Obesity	41 (37.6)	33 (43.4)		8 (24.2)	0.058

SAS (Survey of Autonomic Symptoms); HbA1c (glycated hemoglobin); DM (diabetes mellitus); AC (abdominal circumference); BMI (body mass index); HDL (high density lipoprotein); SBP (systolic blood pressure); DBP (diastolic blood pressure); MS (metabolic syndrome).

^a^ Values expressed as the median (± interquartile range).

^b^ Values expressed as proportion n (%).

^c^ Values expressed as the mean (± standard deviation).

*p values were based on the Mann‒Whitney test (skewed continuous variables), Student’s t test (nonskewed continuous variables), or chi-square test (categorical variables).

** p < 0.05.

# Comparison between participants with SAS ≥ 3 points vs. SAS ≤ 3 points.

Seventy-six patients (69.7%) presented DAN symptoms with a cutoff point ≥ 3 on SAS. The most common symptoms reported were dryness of the oral and ocular mucosa (66.1%) and symptoms of sudomotor dysfunction (answer *yes* to questions 5 and/or 6 and/or 7) were present among 65 (59.6%) participants. The frequency of symptoms according to gender is presented in Table 3. The intensity of symptoms evaluated by Likert scale resulted in a median score of 7.00 points (± 7.5) for males and 12.5 points (± 15.0) for females. The symptoms of leaking urine had the highest median in both men and women (Table 3).

**Table 3:** Symptom frequencies of participants and median of total symptom score (TIS) according to gender

		(%) Affected		Median (± interquartile range) TIS	
SAS questions	**Item**	**Males**	**Females**	**Males**	**Females**
1	Lightheadedness?	44.8	48.8	3.0 (± 1.5)	3.0 (± 3.0)
2	Dry mouth or dry eyes?	55.2	67.5	3.0 (± 1.0)	3.0 (± 2.5)
3	Feet pale or blue?	20.7	25.0	1.5 (± 3.0)	3.0 (± 4.0)
4	Feet colder than the rest of your body?	24.1	37.5	3.0 (± 3.0)	3.0 (± 2.0)
5	Sweating in your feet decreased compared to the rest of your body?	41.4	50.0	2.0 (± 2.0)	3.0 (± 2.0)
6	Sweating in your feet decreased or absent (exercise/hot weather)?	44.8	48.8	1.0 (± 1.0)	3.0 (± 2.0)
7	Sweating in your hands increased compared to the rest of your body?	6.9	16.3	1.0 (± 0.0)	3.0 (± 3.0)
8	Nausea, vomiting, or bloating after eating a small meal?	27.6	45.0	3.0 (± 0.8)	4.0 (± 3.0)
9	Persistent diarrhea?	6.9	17.5	4.0 (± 0.0)	4.5 (± 1.5)
10	Persistent constipation?	10.3	37.5	2.0 (± 4.0)	4.0 (± 2.0)
11	Leaking of urine?	6.9	30.0	4.5 (± 0.0)	5.0 (± 2.0)
12	Difficulty obtaining an erection (men)?	37.9	-	4.0 (± 0.2.0)	
TOTAL				7.00 (± 7.50)	12.50 (± 15.0)

Participants with DAN symptoms showed higher values for age (p = 0.002), HbA1c (p = 0.043), AC (p = 0.019), and BMI (p = 0.013) than the group of subjects without symptoms. There was also a higher frequency of dyslipidemia (p = 0.035) and MS (p < 0.001) among patients with DAN **[**Table 2**].** There was no difference between the sexes (p = 0.295), and participants with T2DM presented a higher frequency of DAN symptoms (78.1%, n = 57) than those with T1DM (52.8%, n = 19) (p = 0.007).

Regarding microvascular complications of DM, DR was present in 39.4%, DKD in 25.2% and DPN in 29.9%. DPN was more frequent in participants with DAN symptoms (p = 0.005) (Table 4). No differences were found for retinopathy nor for DKD.

**Table 4:** Microvascular complications of diabetes comparing the frequency according to the presence of DAN symptoms (SAS ≥ 3) vs. absence of DAN symptoms (SAS < 3)

Characteristics^a^	Overall^b^	SAS ≥ 3^b^	SAS < 3^b^	Value of p ^#*^
DR (n = 66)	26/66 (39.4%)	18/40 (45.0%)	8/26 (30.8%)	0.248
DKD (n = 103)	26/103 (25.2%)	22/72(30.5%)	4/31 (12.9%)	0.059
DPN (n = 87)	26/87 (29.9%)	24/62 (38.7%)	2/25 (8.0%)	0.005**

SAS (Survey of autonomic symptoms); RD (diabetic retinopathy); DKD (diabetes kidney disease); DPN (diabetes polyneuropathy)

^a^ The number of available evaluations for each microvascular complication is indicated on each line

^b^ Values are expressed as proportion n (%)

^#^ Comparison between positive SAS scores (≥ 3 points) and negative SAS scores (≤ 3 points)

*p values were based on the chi-square test

** p < 0.05

We performed a regression model (Table 5) to assess predictive factors for the presence of DAN symptoms (score on SAS ≥ 3). There was an association with age, HbA1c and MS. We found that, in a multiple context, for each increase of 1 year of age and for a 1-unit rise in HbA1c the chance of presenting dysautonomic symptoms increased 1.04-fold and 1.63-fold, respectively. The chance of experiencing dysautonomic symptoms increased by 10.03-fold in the presence of MS.

**Table 5:** Logistic regression for the presence of dysautonomic symptoms (SAS ≥ 3 points) (regression model)

	Β	SE	Wald	df	*P*	Exp(β)	95% C.I. to Exp(β)		
							Bellow	Above	
Age	0.043	0.018	5.452	1	0.020*	1.044	1.007	1.082	
MS	2.306	0.858	7.214	1	0.007*	10.030	1.865	53.954	
HbA1c	0.487	0.224	4.699	1	0.030*	1.627	1.048	2.526	
Constant	7.059	2.124	11.047	1	0.001	0.001			

MS (metabolic syndrome); HbA1c (glycated hemoglobin); β (regression coefficient); SE (standard error); df (degrees of freedom); CI (confidential interval)

* p < 0.05

Neuropad™ was applied to all 65 participants with sudomotor dysfunction (those who answered *yes* to questions 5 and/or 6 and/or 7) and showed abnormal results in 63.1% (n = 41). Among patients with documented DPN (n = 26), Neuropad™ was tested in 19 participants, with an abnormal result in 10 of them (52.6%). Meanwhile, postural hypotension screening, performed in 44 subjects who presented symptoms of lightheadedness/dizziness (which refers to answer *yes* to question 1), was found to be positive in five participants. No significant difference was found when comparing subjects with DAN vs. those without DAN (p = 0.801).

## Discussion

DAN is a complication of DM that is still globally neglected, despite the recognized negative impact on the quality of life of patients [1, 5, 26, 28]. In our study, nearly 70% of participants were found to have the presence of DAN symptoms (SAS ≥ 3 points). The most frequent complaints were “dryness of the oral and ocular mucosa” (Question 2), present in 66.1% of the participants. Other studies confirm “dryness of the oral mucosa” as the most common symptom, while the manifestation of other domains of DAN may vary [6, 21].

The sex distribution showed that 73.4% of patients were females. However, the presence of DAN symptoms was similar between genders (p = 0.295). In our sample, the higher number of females could be explained by more engagement in medical appointments verified in Brazil [29]. Most individuals (66.9%) presented T2DM, and the presence of DAN symptoms was more frequent among them than those with T1DM (p = 0.007). T2DM patients are usually older, and older age is an independent risk factor for DAN [1, 5, 26, 28]. In the present study, for a one-year increase in age, the chance of DAN increased by 1.04 times.

A longer duration of DM is commonly related to symptoms of DAN and is also associated with poorer glycemic control [1, 4, 26, 28]. In our study, DM duration was not associated with the presence of DAN (p = 1.000), but higher HbA1c was found in these patients (p = 0.043). We also observed that for a 1-unit rise in HbA1c, the chance of DAN increased 1.63 times. These data are in line with the most robust evidence for the prevention of DAN in T1DM, with a focus on CAN: adequate glycemic control, as demonstrated in the Diabetes Control and Complications (DCCT) and the Epidemiology of Diabetes Interventions and Complications (EDIC) studies, reduced the risk of CAN by 45% and 31%, respectively [30–33].

The frequency of MS was higher among patients with the presence of DAN symptoms (p = 0.001), and this condition impressively increased the chance of having DAN by 10.030 times. Higher BMI was also present in subjects with the presence of DAN symptoms (p = 0.001). None of the previous studies with SAS, including the pioneer one by Zilliox et al. [6], have searched for connection with MS or its components [21, 34].

Indeed, several previous reports have demonstrated an association between MS and obesity with DPN as well as with CAN [1, 5, 30, 35–37]. This is relevant, as CAN is the most studied form of DAN [1, 4, 5, 12]. In a Chinese series, 2092 patients with MS were evaluated, and there was a 24% prevalence of CAN [37], but no data were reported for other forms of DAN. Our data reinforce the potential of a multifactorial approach, including lifestyle changes, to prevent dysautonomic symptoms. This fact was well demonstrated in the STENO-2 study [38], in which patients with T2DM underwent a 7-year cardiovascular intervention (control of hypertension, dyslipidemia, and blood glucose), resulting in a 68% reduction in the risk progression of CAN. Again, no mention to other forms of DAN was referred, even in this important reference study.

Among the microvascular complications, which are predictive factors for DAN [1, 4, 5, 28], DR and DKD were not associated with the presence of DAN. On the other hand, DPN was more frequent when DAN symptoms were present (p = 0.005). In the Zilliox et al. study [6], there was also a predominance of DPN among participants with DAN symptoms, which also points out the connection of DPN and DAN symptoms.

Sudomotor dysfunction is one of the manifestations of peripheral dysautonomia and results from impaired innervation of sweat glands under the control of post ganglionic cholinergic sympathetic small type C fibers, which is associated with DPN, since hypohidrosis/anhidrosis are indicative of early preulcerative foot lesions [7–9]. In addition, previous studies report the presence of sudomotor dysfunction as a predictive factor for CAN [5, 39], which may be broadly screened, since the high risk of mortality conferred by the presence of CAN has been clearly demonstrated [35, 36, 40].

There are several tools for assessing sudomotor dysfunction that vary in complexity and accuracy [4, 7–9]. Among them, the Neuropad™ test has already been widely studied in Europe as a screening instrument for sudomotor dysfunction as well as for DPN and CAN [5, 7, 8, 25].

In the study by Gómez-Banoy et al., 66.6% of patients with DPN presented an abnormal Neuropad™ result [41]. The study by Mendevil et al., also from Colombia, analyzed 154 patients with DM, showing that 67.5% had an abnormal Neuropad™ test, and higher neuropathic symptom scores (Michigan Neuropathy Disability Score – MNDS and Total Symptoms Score – TSS) were found in those with an abnormal test [42].

In our sample, Neuropad™ was abnormal in the majority of participants with sudomotor dysfunction symptoms (63.1%) and documented DPN (52.6%). This suggests a combination of sudomotor dysfunction and DPN, reinforcing the association between DAN and DPN in this scenario of peripheral dysautonomic impairment [8, 9, 41, 42].

In the Korean study by Kim et al. [34], SAS was applied to 76 people with DM, and a statistically significant association was verified between the presence of lightheadedness/dizziness (Question 1) and orthostatic hypotension. In a study carried out with 396 patients with diabetes, the symptom of postural lightheadedness/dizziness was present in 10.4% (n = 39) of the participants, but no association was found with orthostatic hypotension [39]. In our study, 40% of patients (n = 44) reported dizziness, but a higher frequency of diagnosis of postural hypotension in patients with the presence of DAN was not found (p = 0.801). “The association between lightheadedness/dizziness and postural hypotension have been previously shown to be present [34] or not [39]. Postural hypotension is a late finding of cardiac dysautonomia alluding to severe CAN [1, 4, 5, 28] that would need to be confirmed by cardiac specific tests, which was not in the scope of this study. A small number (only five patients) had postural hypotension. These points might explain our findings.”

This study inserts Brazil among the very few countries that have tested SAS as an instrument to early identify symptoms of DAN among people with diabetes. The application of the Brazilian version of SAS and the joint use of the Neuropad™ adhesive proved to be promising screening tools since they are simple tests and require a short time to be applied.

Therefore, to our knowledge, this is the first study that applied SAS through an app. The Brazilian version of SAS (QSA) was developed to be used in computers and smart mobile phones. This strategy facilitated data collection, considering its simplicity, objectivity, and agility, in addition to allowing dispensing the use of paper and contributing to the preservation of the environment. Currently, when telemedicine has become an important means to extend knowledge and access [43, 44], mainly due to the COVID pandemic [45, 46], the use of SAS through an app might turn into a way to spread DAN evaluation, especially in the context of public health, where material resources are more restricted.

This research presented some limitations. In the context of the COVID-19 pandemic, patients with noncommunicable chronic diseases have faced barriers to access to either routine clinical visits or other specialties and this was also happened in Brazil [47]. This imposed limitations to this study, contributing to not enroll more patients and enlarge screening data for microvascular complications, mainly in the primary care. The latter might explain the absence of a relationship between DR and CKD with dysautonomic symptoms.

## Conclusions

The high prevalence of DAN symptoms found in this study highlights the importance of screening for this complication. MS increased the risk of DAN in a robust manner. The use of SAS through an app was shown to be a practical, easy-to-use and short time demand tool that potentially contributes to circumvent DAN underdiagnosis. The combined use of SAS and Neuropad™ seems to be useful to ascertain the presence of peripheral small fiber autonomic dysfunction and to identify preulcerative lesions.

## Electronic supplementary material

Below is the link to the electronic supplementary material.

Supplementary Material 1

## Data Availability

The datasets used and/or analyzed during the current study are available from the corresponding author on reasonable request.

## Abbreviations

DAN: Diabetic autonomic neuropathy
CAN: Cardiovascular autonomic neuropathy
SAS: Survey of Autonomic Symptoms
ASP: Autonomic Symptom Profile
COMPASS: Composite Autonomic Symptom Scale
CASS: Composite Autonomic Scoring Scale
QSART: Quantitative Sudomotor axonal Reflex
DPN: Diabetic peripheral neuropathy
DM: Diabetes mellitus
DR: Diabetic retinopathy
DKD: Diabetes kidney disease
UENDO-POLO-HRT: Endocrinology Unit of the Taguatinga Regional Hospital/Research Center
T1DM: Type 1 diabetes
T2DM: Type 2 diabetes
AC: Abdominal circumference
BMI: Body mass index
MS: Metabolic syndrome
IDF: International Diabetes Federation
ACR: Albumin/creatinine ratio
PAD: Peripheral arterial disease
NDS: Neuropathy Disability Score
IWGDF: Working Group on the Diabetic Foot
TIS: Total symptom score (TIS).
QSA: Questionário de Sintomas Autonômicos (Portuguese/Brazilian Versions of SAS)
PWA: Progressive Web App (PWA)
API: Applications Protocol Interface
PHP: Developed in Hypertext Preprocessor
MySQL: Management system based on a query language
SBP: Systolic blood pressure
DBP: Diastolic blood pressure
SD: Standard deviations
SPSS: Package for the Social Sciences
CAAE: Certificate of Presentation for Ethical Appreciation
DCCT: Diabetes Control and Complications
EDIC: Epidemiology of Diabetes Interventions and Complications
