# Supplementary material for: Autonomic neuropathic symptoms in patients with diabetes: practical tools for screening in daily routine
Source: Diabetol Metab Syndr. 2023 Apr 26;15:83. doi: 10.1186/s13098-023-01036-7 (PMC10130803; doi:10.1186/s13098-023-01036-7)
Supplement: Supplementary file 1 — Supplementary Material 1 [file 13098_2023_1036_MOESM1_ESM.pdf]

**Questionário de sintomas autonômicos (QSA)**	**1. Você tem algum desses sintomas:**<br><br>**Sim = 1**<br><br>**Não = 0**	**2. Se a resposta é SIM, quanto esse sintoma incomoda você?**<br><br>***1=quase nada***<br><br>***2=bem pouco***<br><br>***3=pouco***<br><br>***4=bastante 5=muito***

1. Você sente tontura?	1	0	1	2	3	4	5
2. Você sente a boca ou os olhos secos?	1	0	1	2	3	4	5
3. Seus pés são pálidos ou arroxeados?	1	0	1	2	3	4	5
4. Você costuma sentir seus pés mais frios do que o restante do corpo?	1	0	1	2	3	4	5
5. Você sua (transpira) menos nos pés do que no restante do corpo?							
6. Você sua (transpira) pouco ou nada nos pés (por exemplo, depois de fazer exercício físico ou em dias quentes)?	1	0	1	2	3	4	5
7. Você sua (transpira) mais nas mãos do que no restante do corpo?	1	0	1	2	3	4	5
8. Você tem náusea, vontade de vomitar ou sente inchaço no abdome após fazer uma pequena refeição?	1	0	1	2	3	4	5
9. Você tem diarreia (mais de três evacuações ao dia) por vários dias seguidos?	1	0	1	2	3	4	5
10. Você tem o intestino preso (menos de uma evacuação a cada dois dias) que dura vários dias?	1	0	1	2	3	4	5
11. Você tem incontinência urinária (não segura a urina)?	1	0	1	2	3	4	5
12. Você tem dificuldade de ter ereção **(SOMENTE PARA SEXO MASCULINO)**	1	0	1	2	3	4	5
